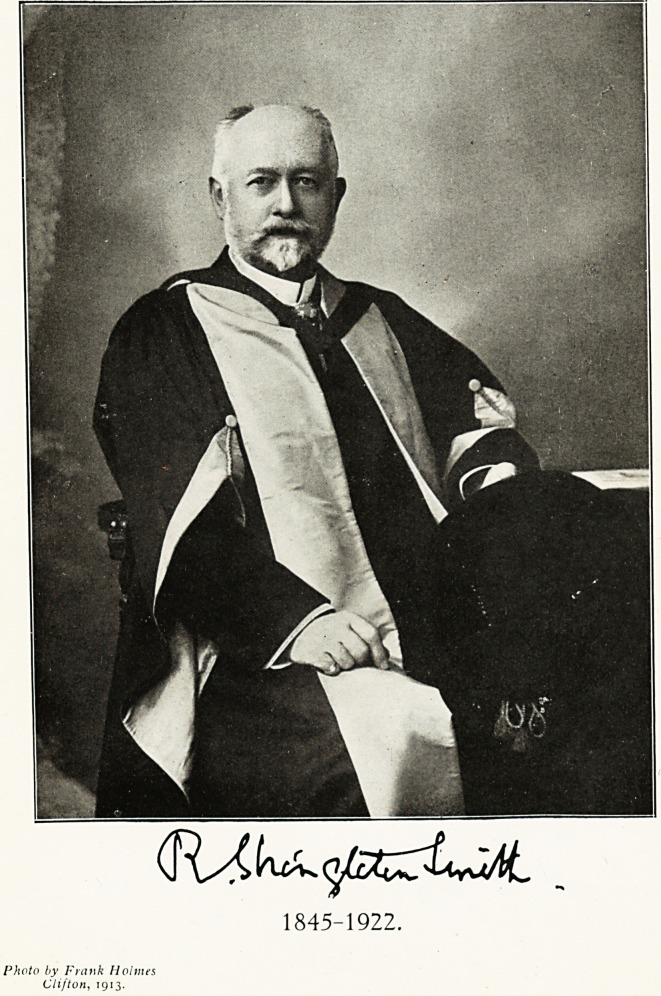# Robert Shingleton Smith

**Published:** 1922-06

**Authors:** 


### ROBERT SHINGLETON SMITH,

### M.D., B.Sc., F.R.C.P.

1845—1922.

---

Our portrait of Dr. R. Shingleton Smith was taken shortly after he had resigned the Editorship of this *Journal*, after guiding its destinies for twenty years.

In the Obituary Notice, his career and his services to the medical profession are related. Here the Editorial Committee, sensible of the privilege of their association with the late Dr. Shingleton Smith as Editor, and mourning this distinguished friend in whose footsteps they have been proud to tread, desire to record their deep sense of bereavement.

## Figures and Tables

**Figure f1:**